# Supplementary figures and images for: Distinct regions of H. pylori’s bactofilin CcmA regulate protein–protein interactions to control helical cell shape
Source: eLife. 2022 Sep 8;11:e80111. doi: 10.7554/eLife.80111 (PMC9507126; doi:10.7554/eLife.80111)

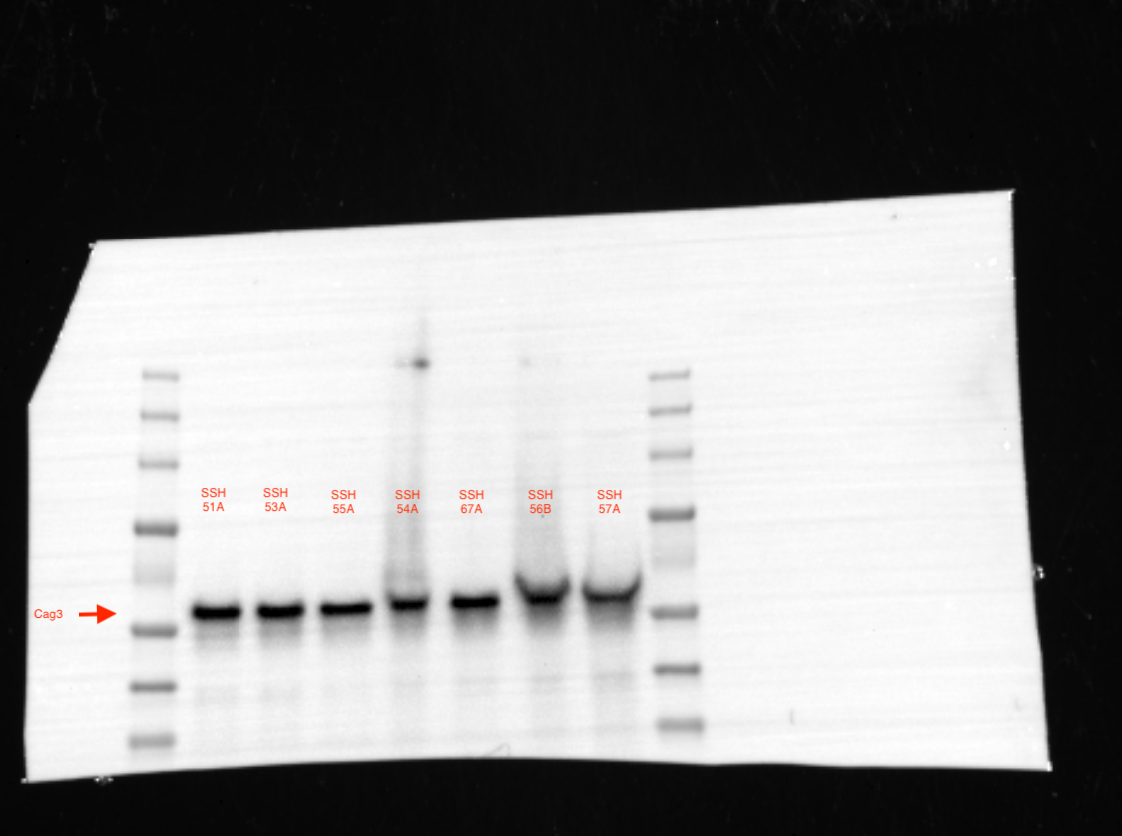

Supplement: Figure 2—source data 1. [file elife-80111-fig2-data1.zip › Figure_2_source_data/Cag3.tif]

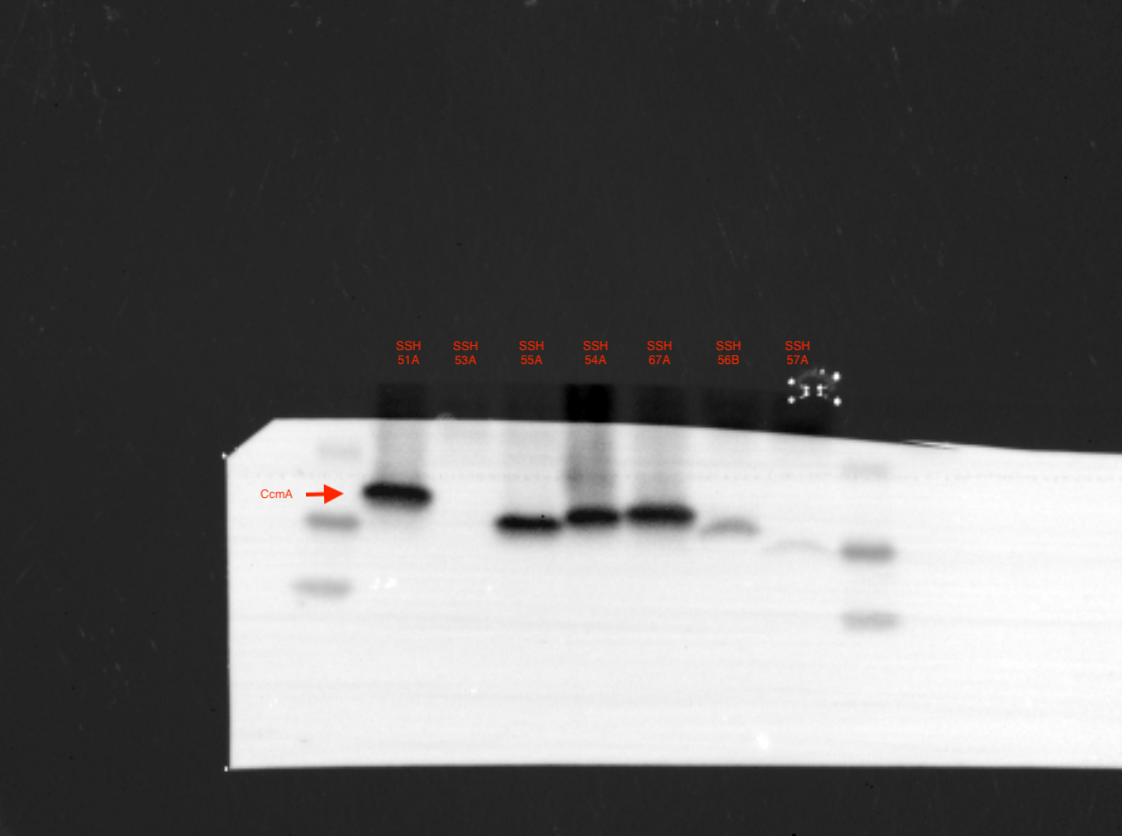

Supplement: Figure 2—source data 1. [file elife-80111-fig2-data1.zip › Figure_2_source_data/CcmA.tif]

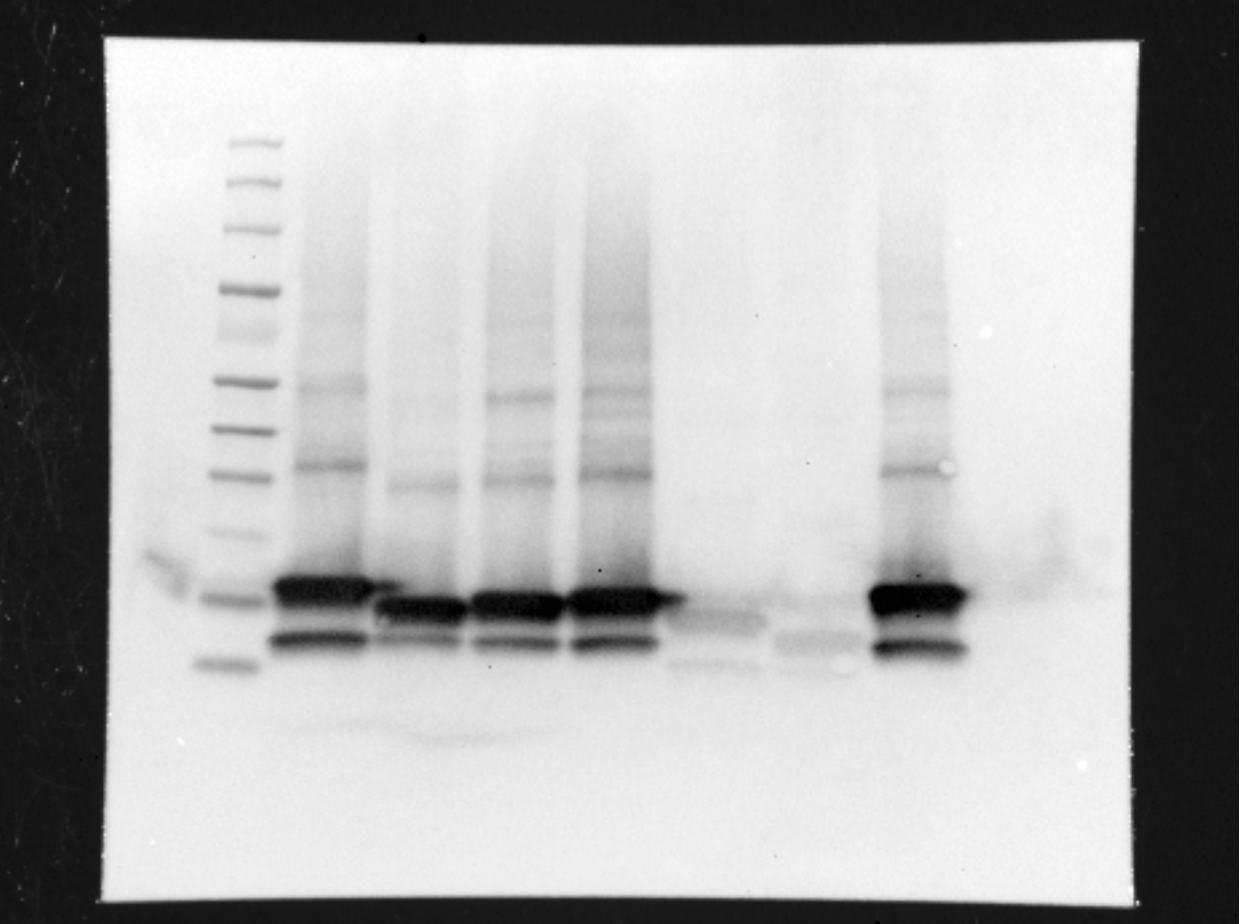

Supplement: Figure 2—figure supplement 1—source data 1. [file elife-80111-fig2-figsupp1-data1.zip › Figure_2_supp_1_source_data/CcmA.tif]

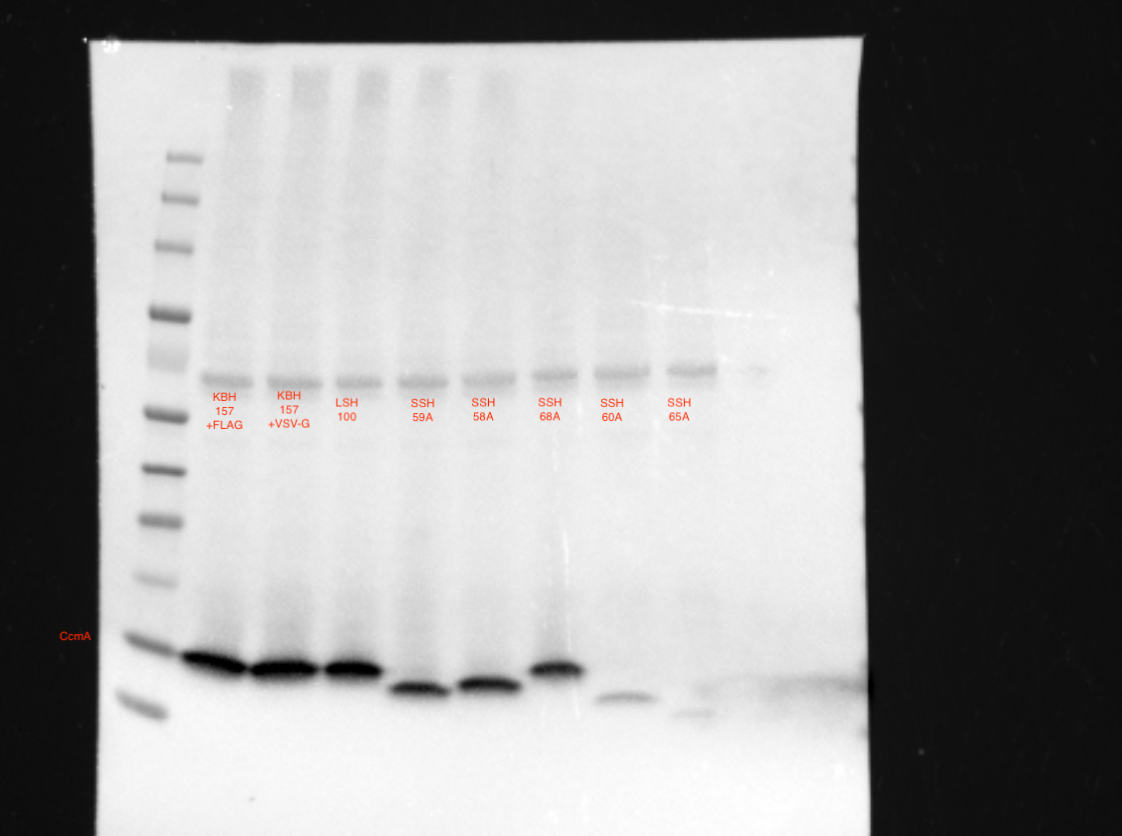

Supplement: Figure 5—source data 1. [file elife-80111-fig5-data1.zip › Figure_5_source_data/Csd5_CoIPs/inputs_CcmA.tif]

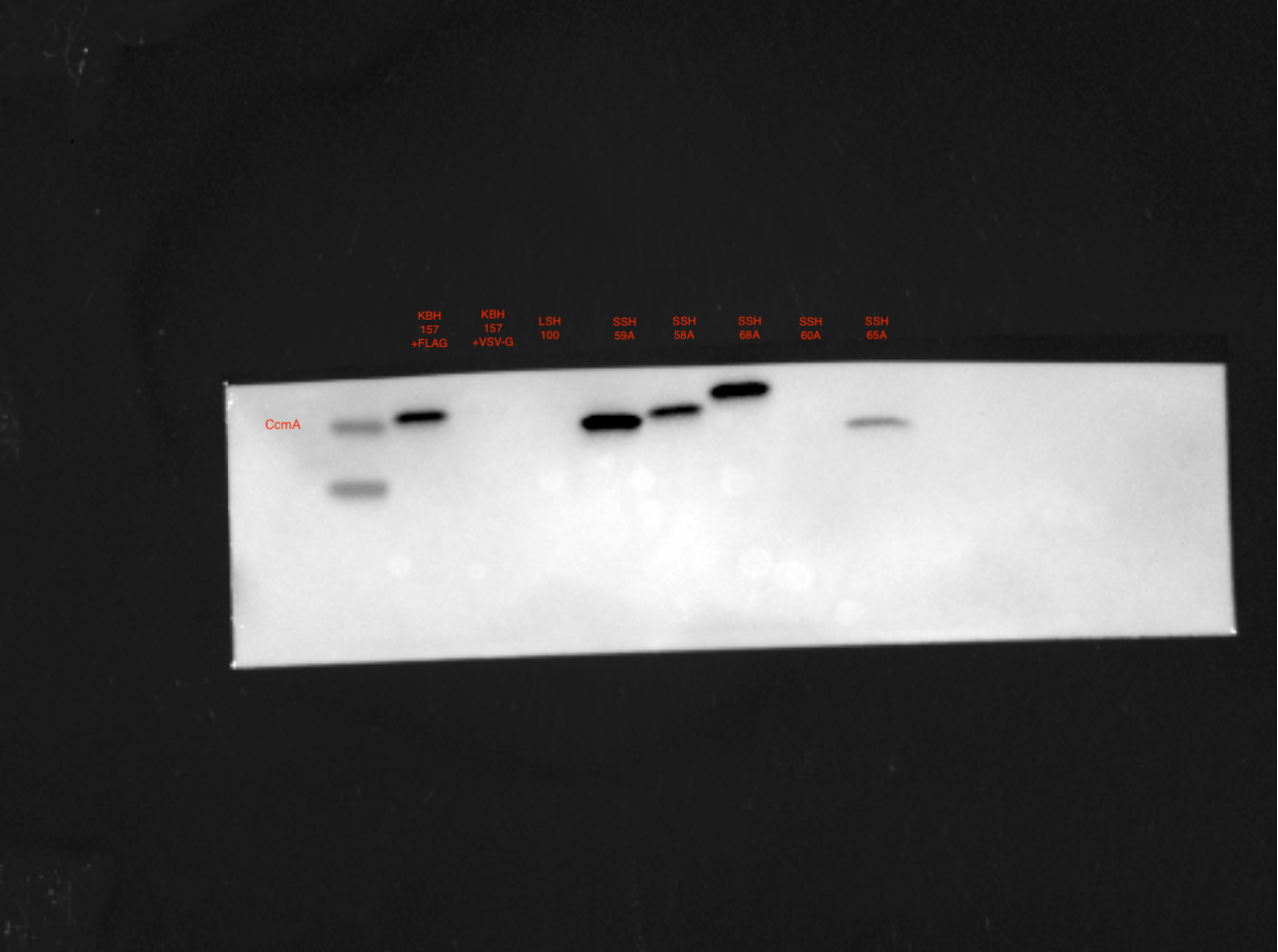

Supplement: Figure 5—source data 1. [file elife-80111-fig5-data1.zip › Figure_5_source_data/Csd5_CoIPs/IPs_CcmA.tif]

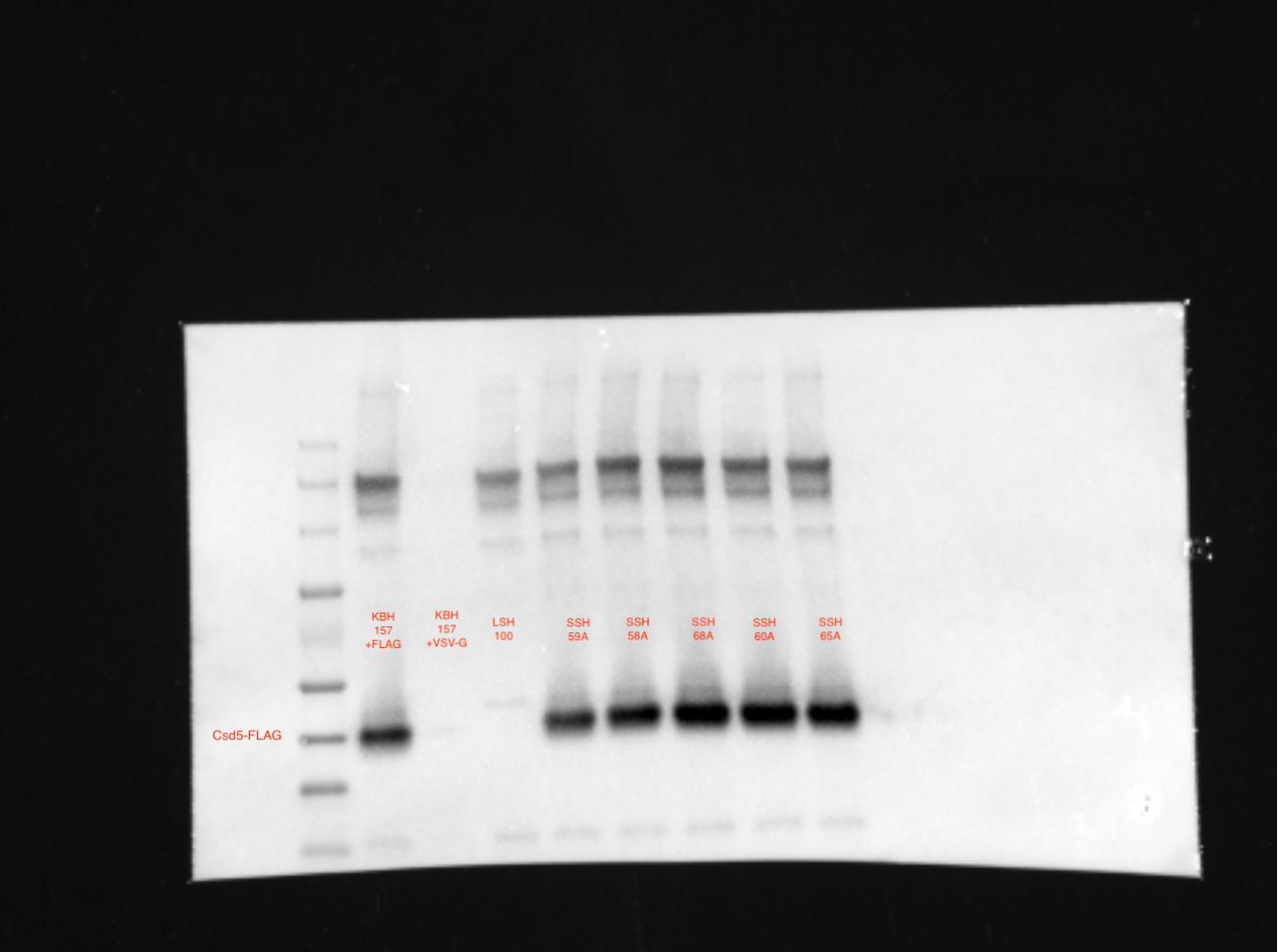

Supplement: Figure 5—source data 1. [file elife-80111-fig5-data1.zip › Figure_5_source_data/Csd5_CoIPs/IPs_FLAG.tif]

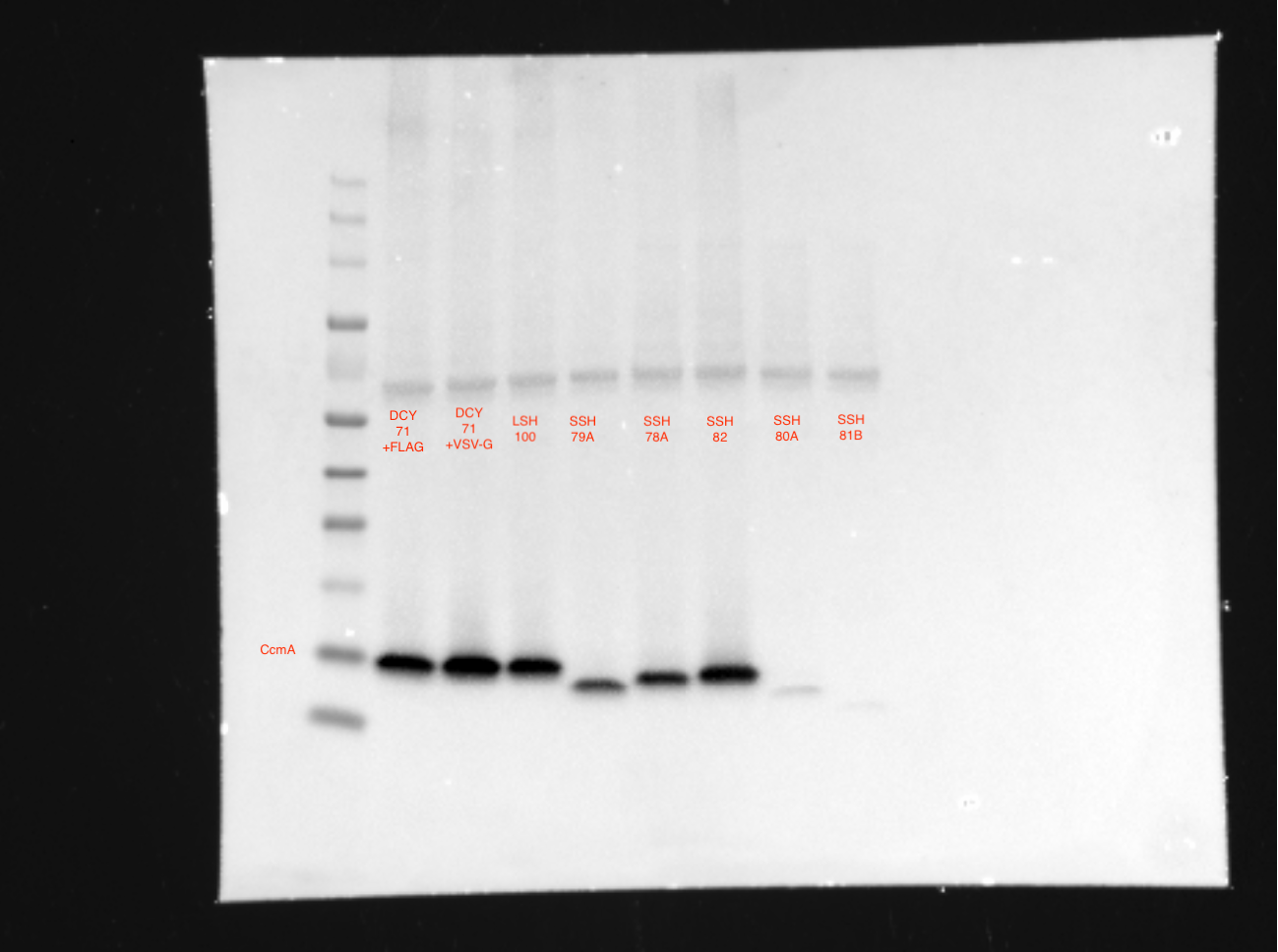

Supplement: Figure 5—source data 1. [file elife-80111-fig5-data1.zip › Figure_5_source_data/Csd7_CoIPs/csd7-FLAG IP inputs ladder only+csd7-FLAG IP inputs anti ccmA 2.tif]

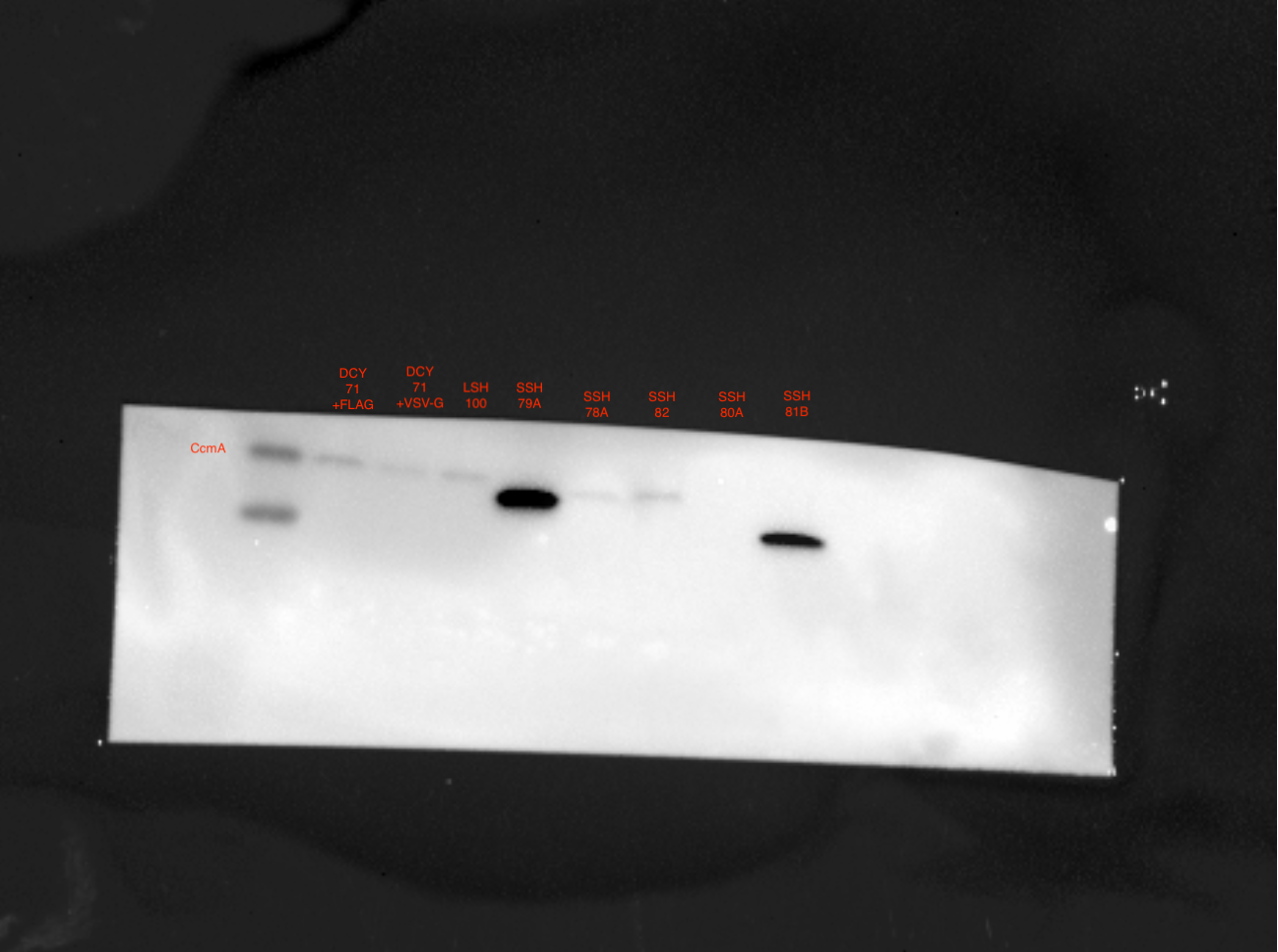

Supplement: Figure 5—source data 1. [file elife-80111-fig5-data1.zip › Figure_5_source_data/Csd7_CoIPs/csd7-FLAG IP IPs bottom ladder only+csd7-FLAG IP IPs bottom anti ccmA.tif]

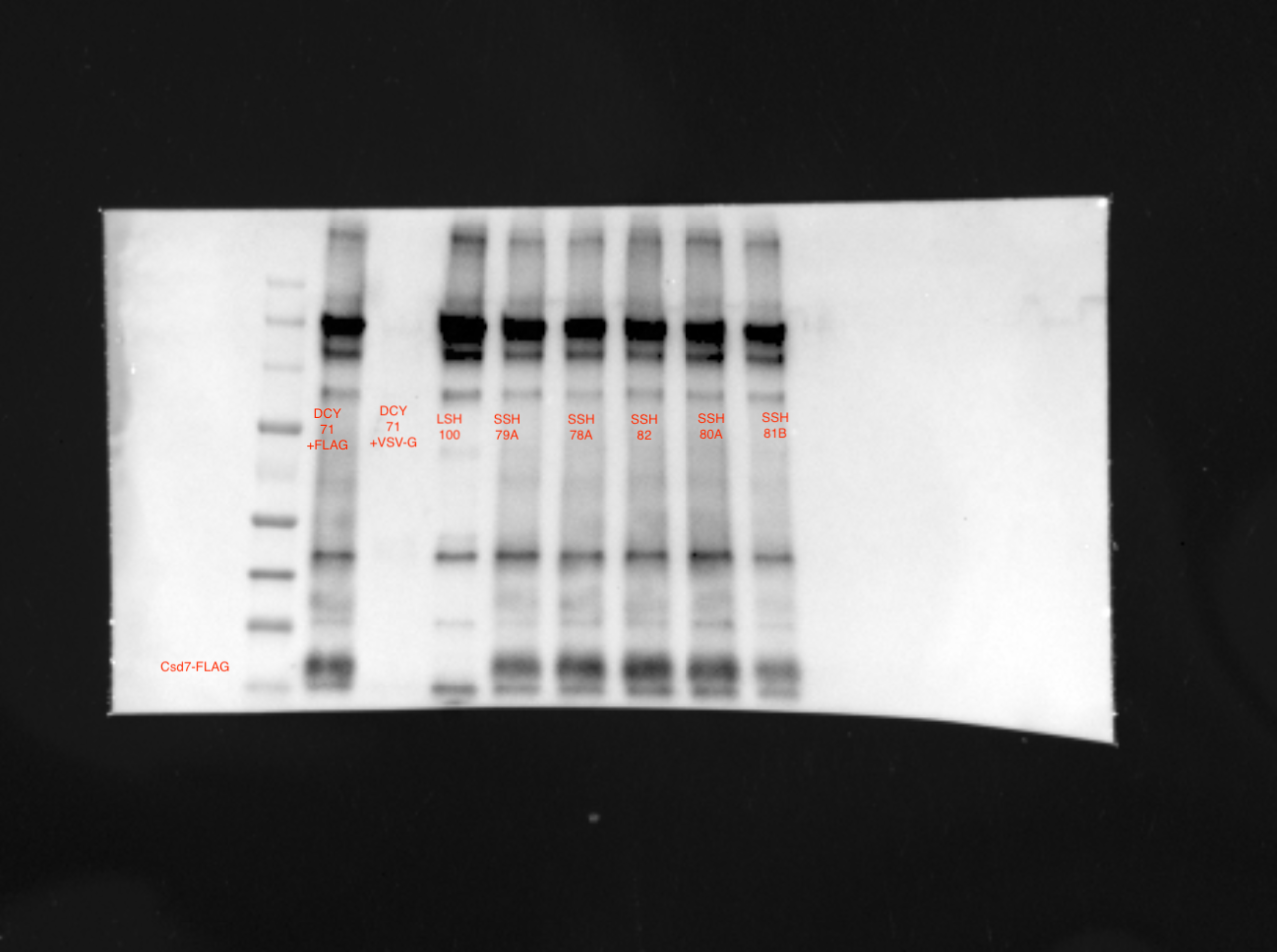

Supplement: Figure 5—source data 1. [file elife-80111-fig5-data1.zip › Figure_5_source_data/Csd7_CoIPs/csd7-FLAG IP IPs top ladder only+csd7-FLAG IP IPs top anti FLAG.tif]

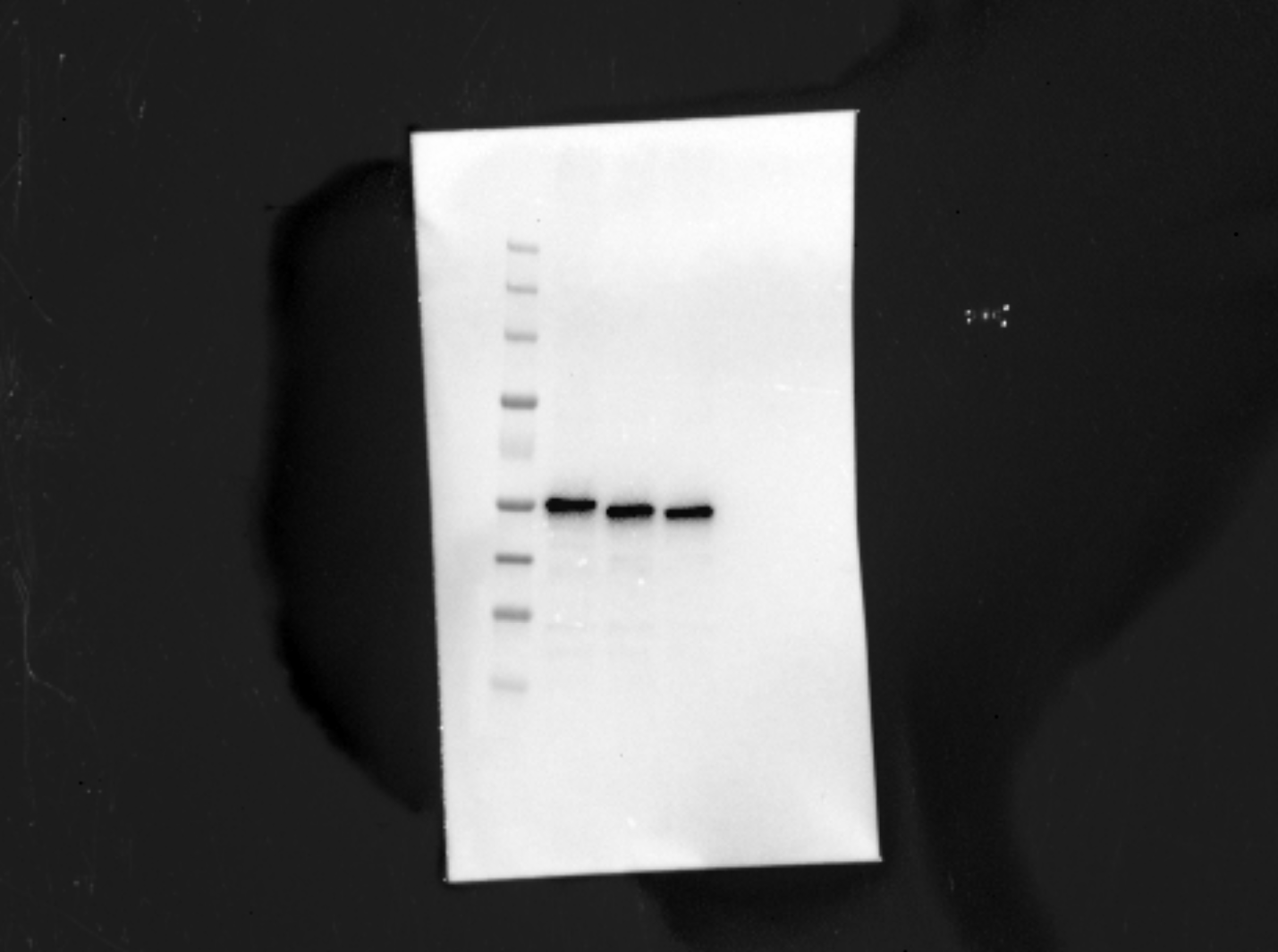

Supplement: Figure 5—figure supplement 1—source data 1. [file elife-80111-fig5-figsupp1-data1.zip › Figure_5_supp_1_source_data/Inputs_HaloTag.tif]

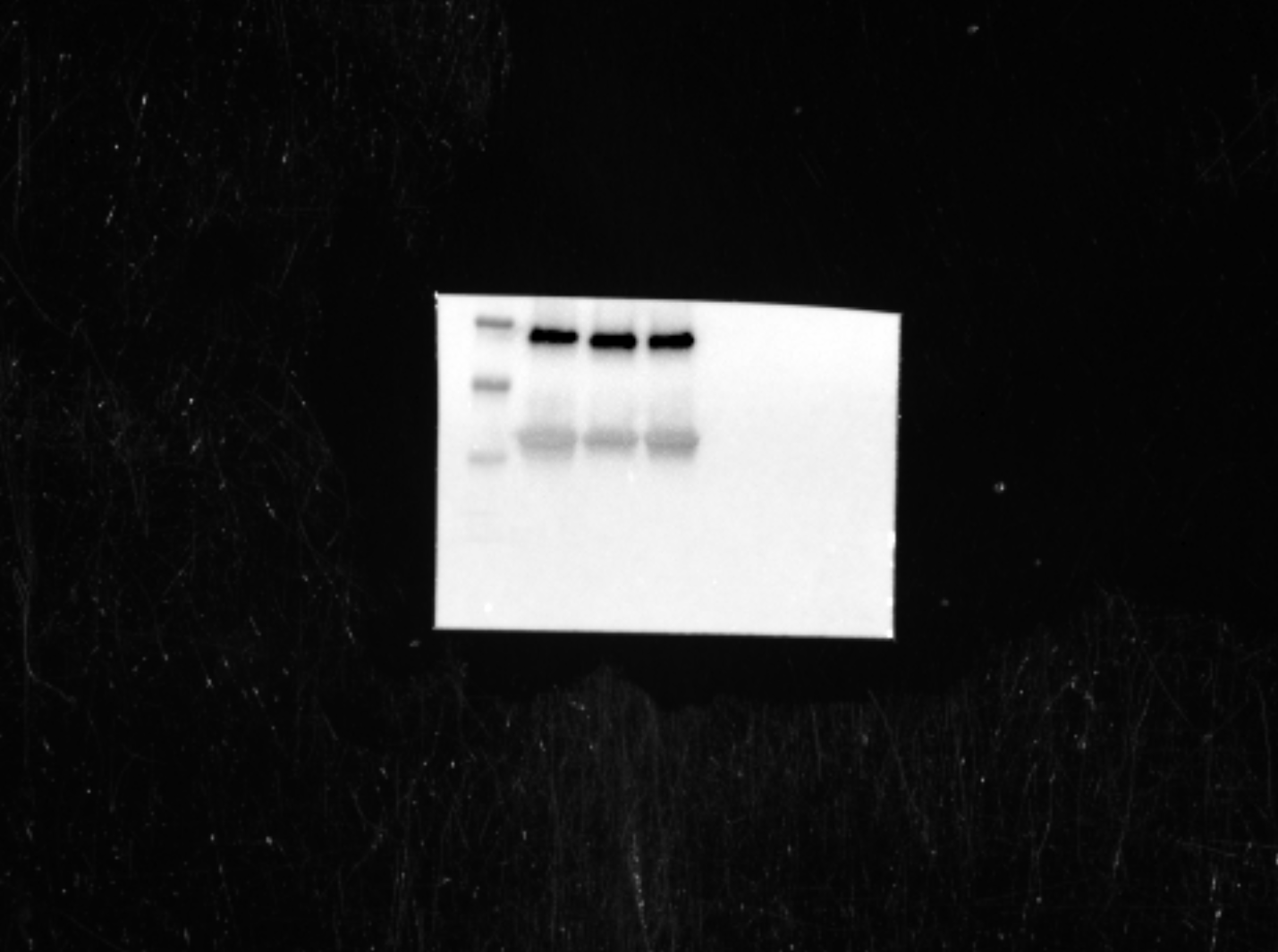

Supplement: Figure 5—figure supplement 1—source data 1. [file elife-80111-fig5-figsupp1-data1.zip › Figure_5_supp_1_source_data/IPs_FLAG.tif]

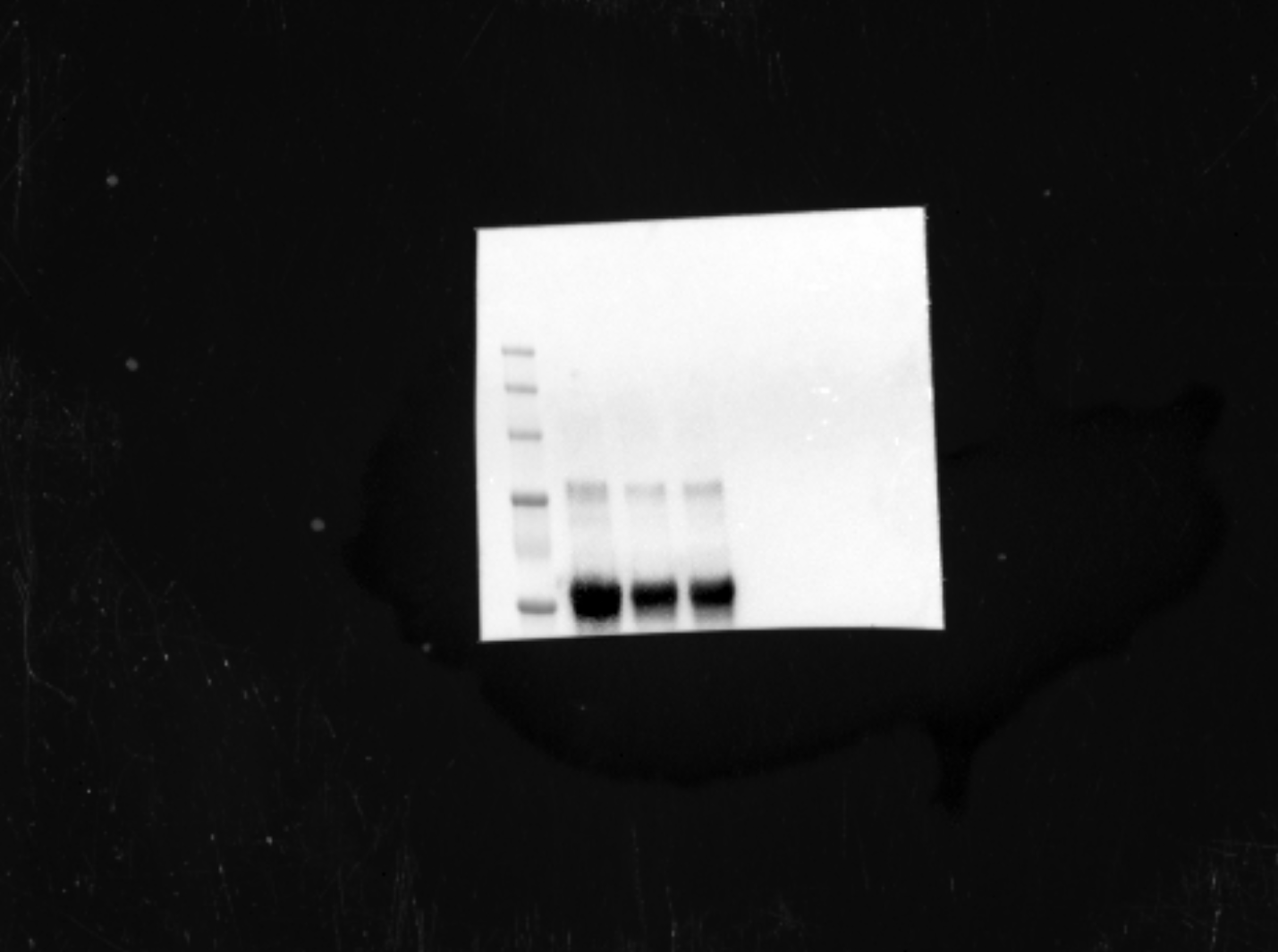

Supplement: Figure 5—figure supplement 1—source data 1. [file elife-80111-fig5-figsupp1-data1.zip › Figure_5_supp_1_source_data/IPs_HaloTag.tif]

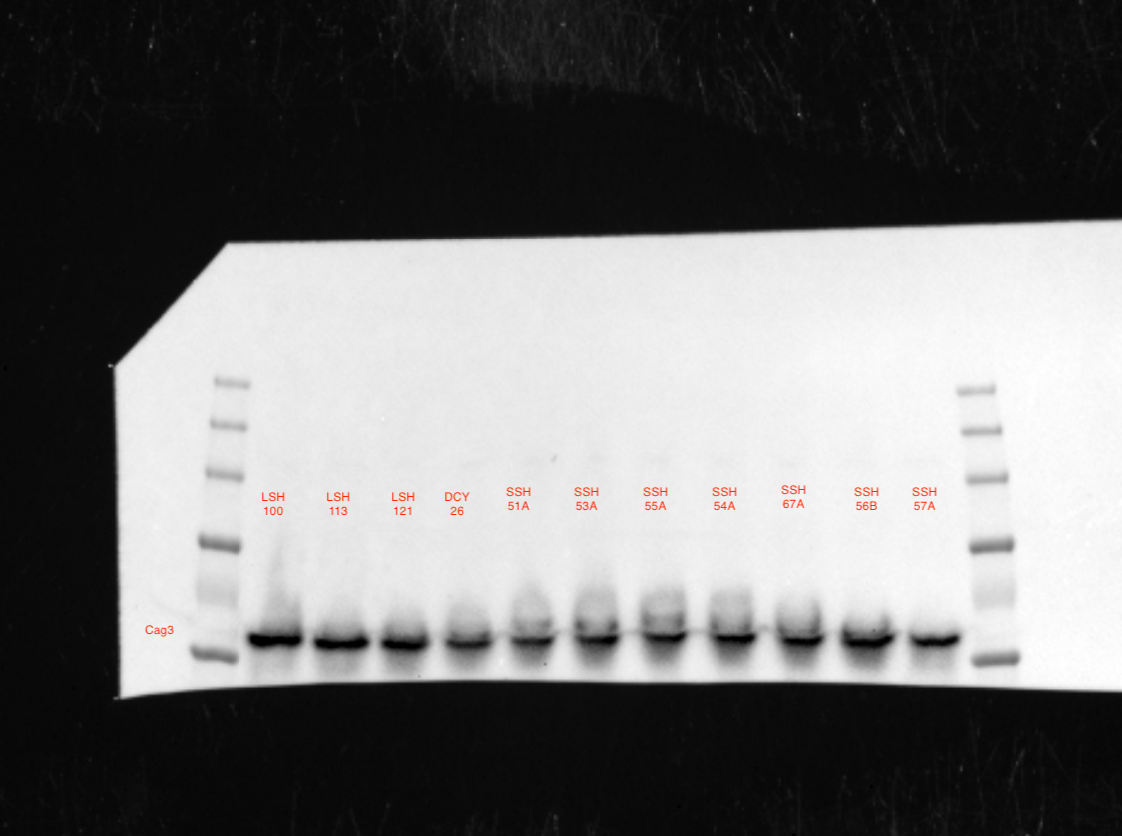

Supplement: Figure 6—source data 1. — In addition, raw, unedited Western blots probed with ɑ-CcmA and ɑ-FLAG, ɑ-Cag3 and ɑ-Csd1 antibodies to detect CcmA, Csd1, and Csd7-FLAG in co-immunoprecipitation experiments. [file elife-80111-fig6-data1.zip › Figure_6_source_data/Panel_A/Cag3.tif]

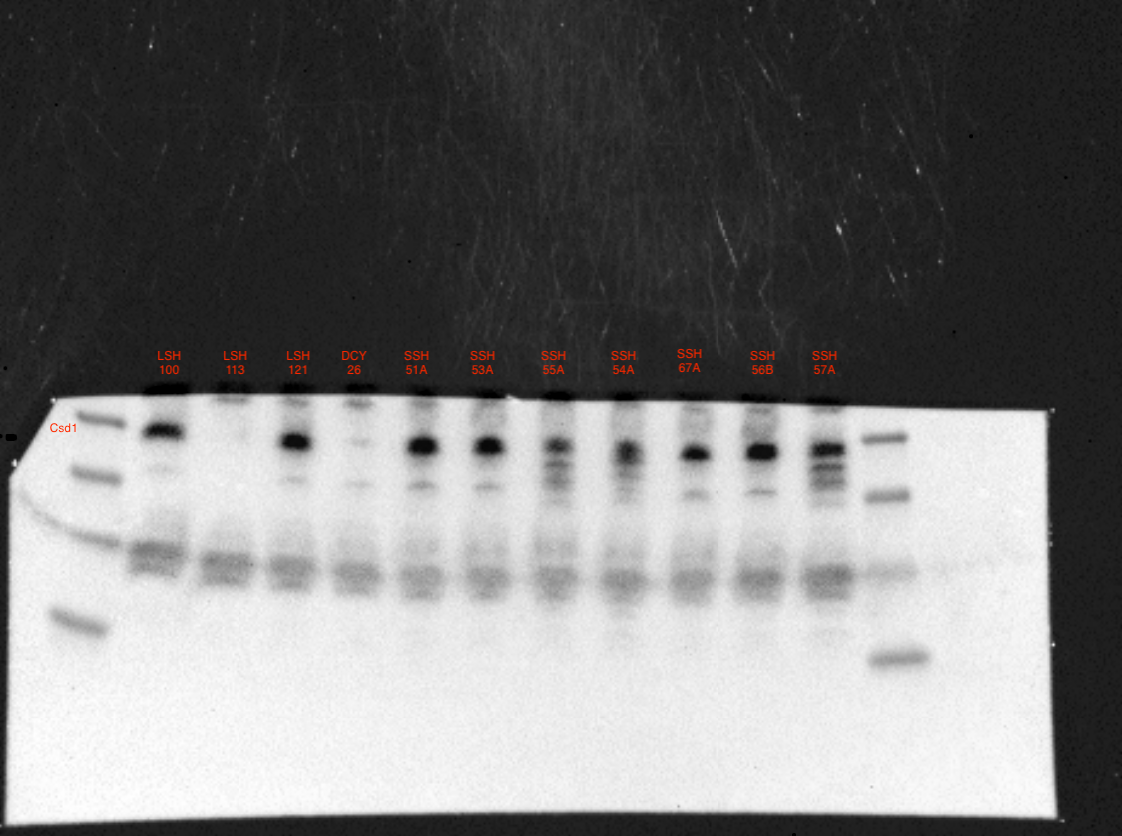

Supplement: Figure 6—source data 1. — In addition, raw, unedited Western blots probed with ɑ-CcmA and ɑ-FLAG, ɑ-Cag3 and ɑ-Csd1 antibodies to detect CcmA, Csd1, and Csd7-FLAG in co-immunoprecipitation experiments. [file elife-80111-fig6-data1.zip › Figure_6_source_data/Panel_A/Csd1.tif]

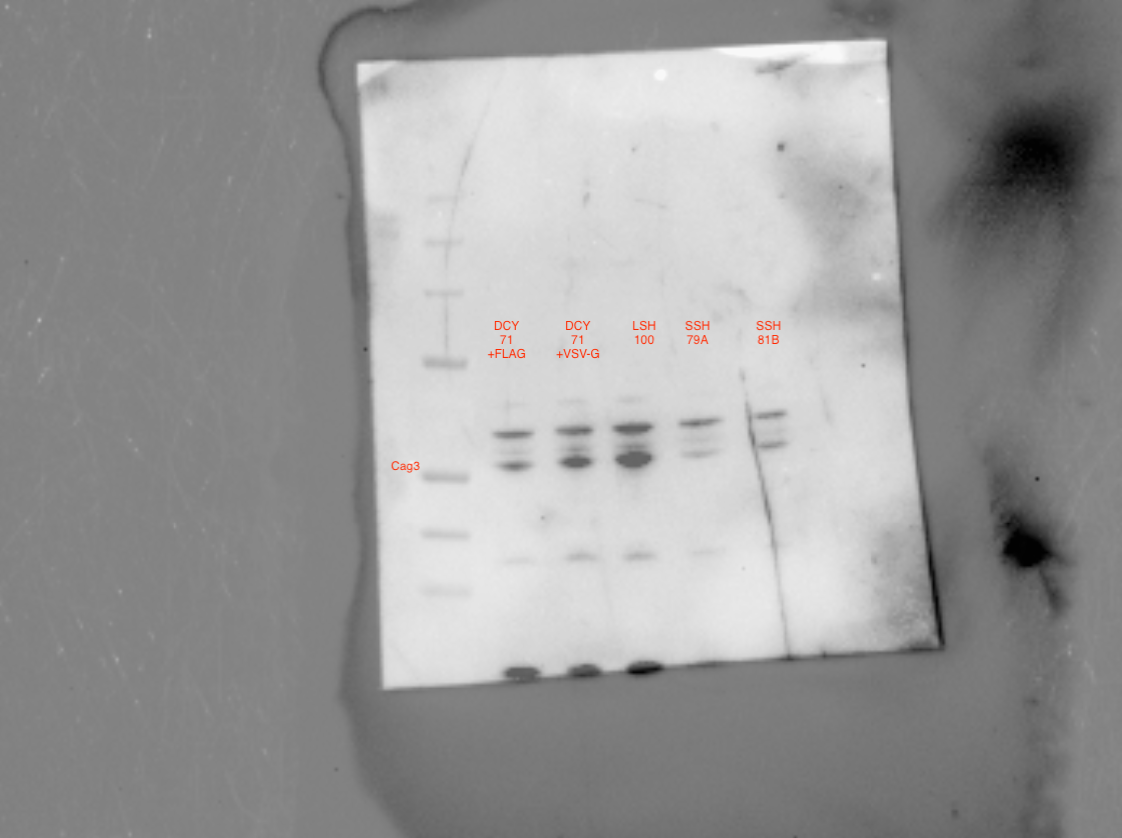

Supplement: Figure 6—source data 1. — In addition, raw, unedited Western blots probed with ɑ-CcmA and ɑ-FLAG, ɑ-Cag3 and ɑ-Csd1 antibodies to detect CcmA, Csd1, and Csd7-FLAG in co-immunoprecipitation experiments. [file elife-80111-fig6-data1.zip › Figure_6_source_data/Panel_C/input_Cag3.tif]

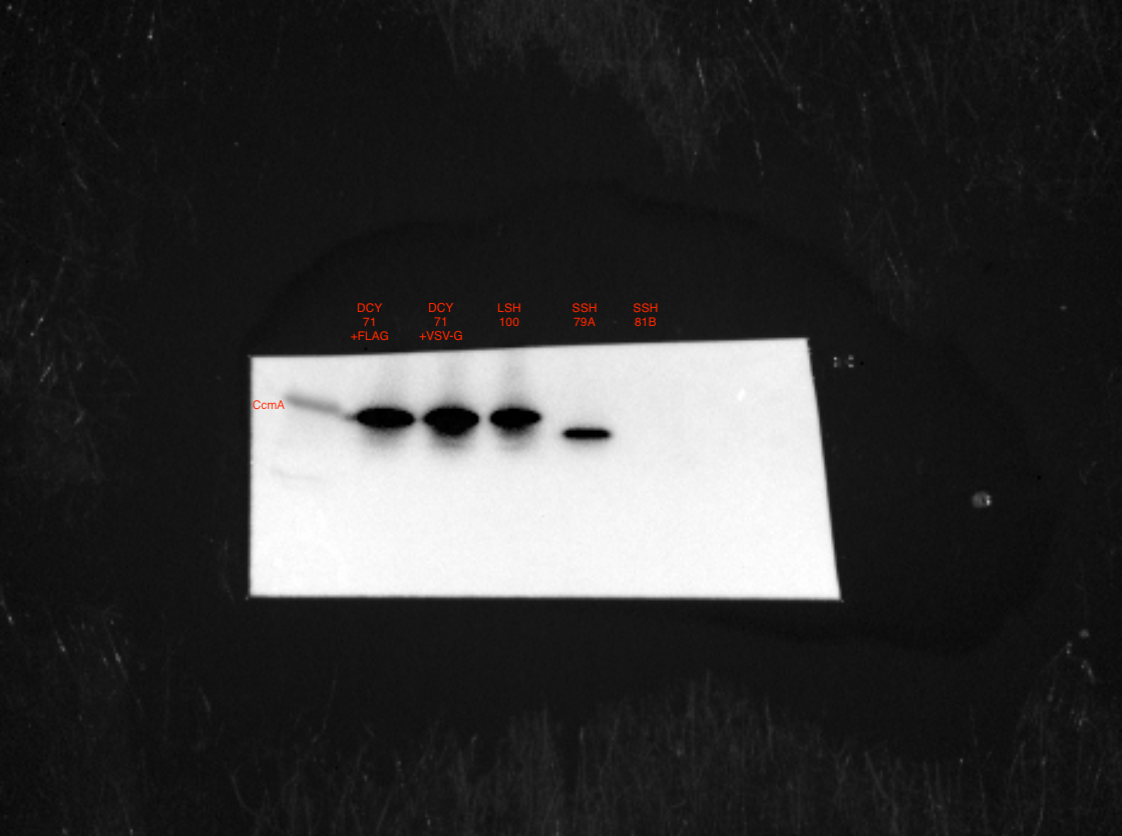

Supplement: Figure 6—source data 1. — In addition, raw, unedited Western blots probed with ɑ-CcmA and ɑ-FLAG, ɑ-Cag3 and ɑ-Csd1 antibodies to detect CcmA, Csd1, and Csd7-FLAG in co-immunoprecipitation experiments. [file elife-80111-fig6-data1.zip › Figure_6_source_data/Panel_C/input_CcmA.tif]

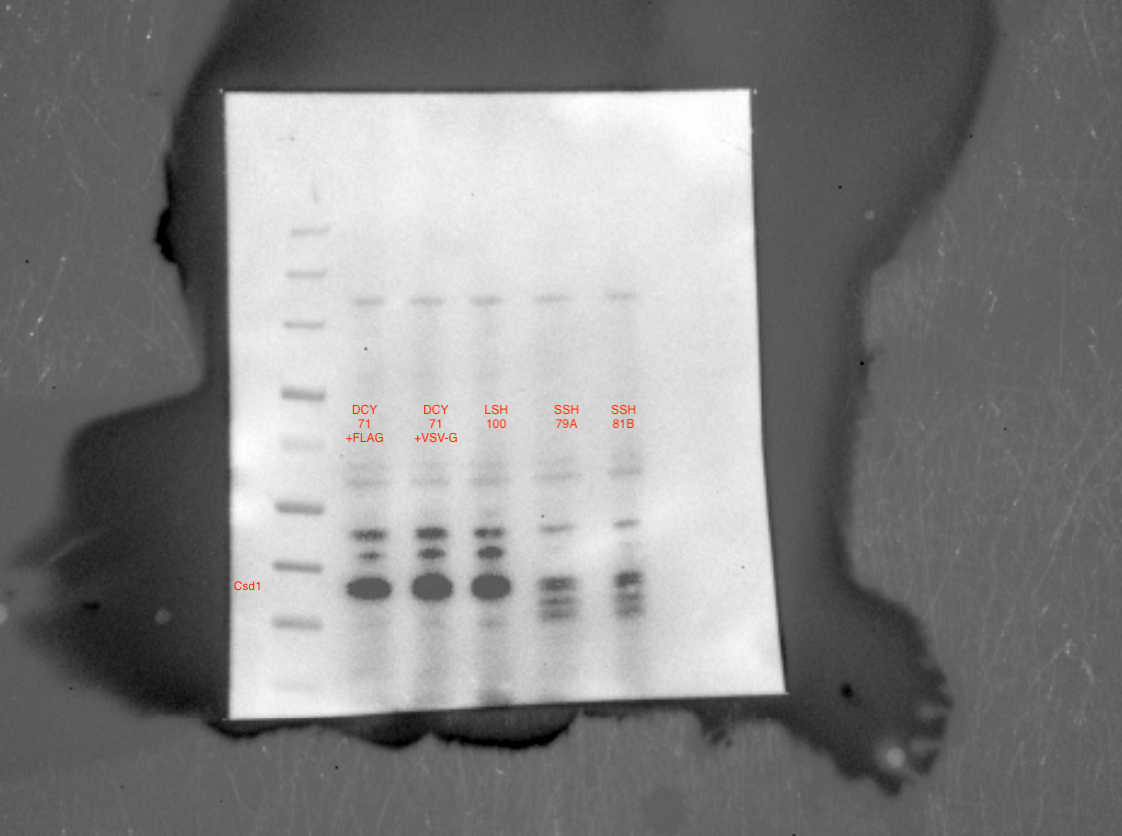

Supplement: Figure 6—source data 1. — In addition, raw, unedited Western blots probed with ɑ-CcmA and ɑ-FLAG, ɑ-Cag3 and ɑ-Csd1 antibodies to detect CcmA, Csd1, and Csd7-FLAG in co-immunoprecipitation experiments. [file elife-80111-fig6-data1.zip › Figure_6_source_data/Panel_C/input_Csd1.tif]

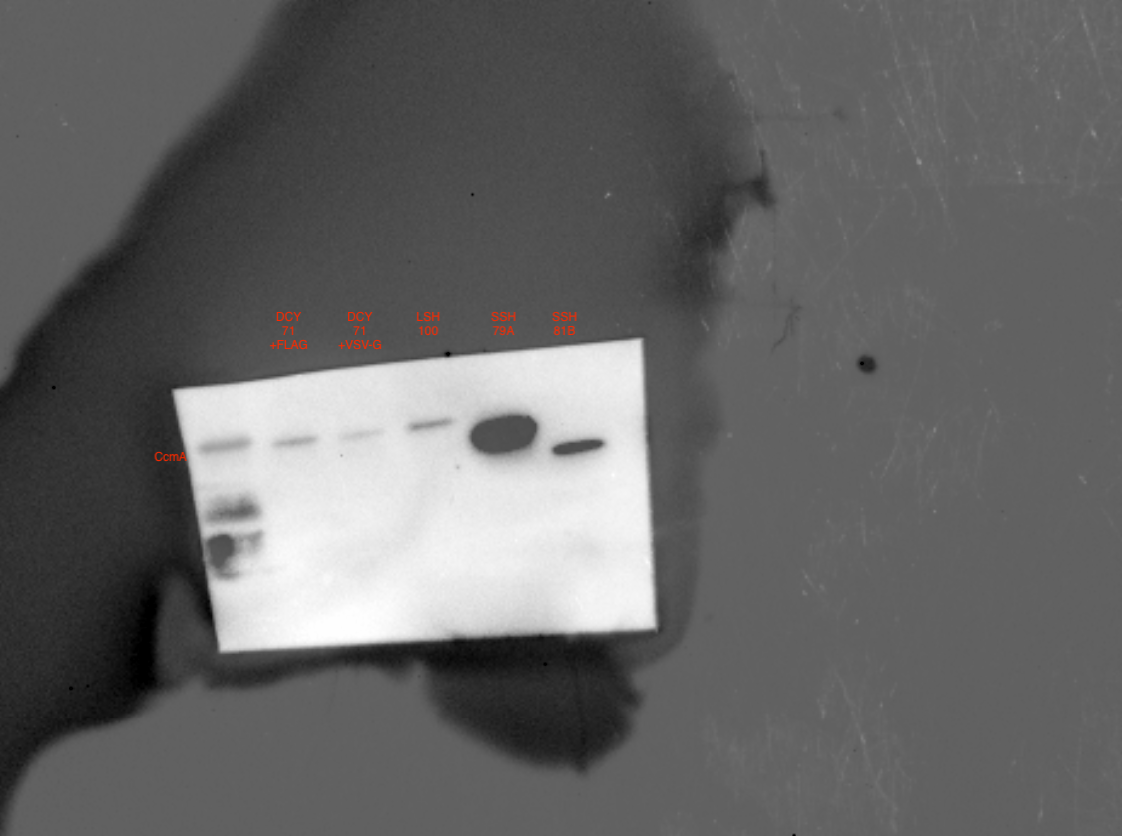

Supplement: Figure 6—source data 1. — In addition, raw, unedited Western blots probed with ɑ-CcmA and ɑ-FLAG, ɑ-Cag3 and ɑ-Csd1 antibodies to detect CcmA, Csd1, and Csd7-FLAG in co-immunoprecipitation experiments. [file elife-80111-fig6-data1.zip › Figure_6_source_data/Panel_C/IP_CcmA.tif]

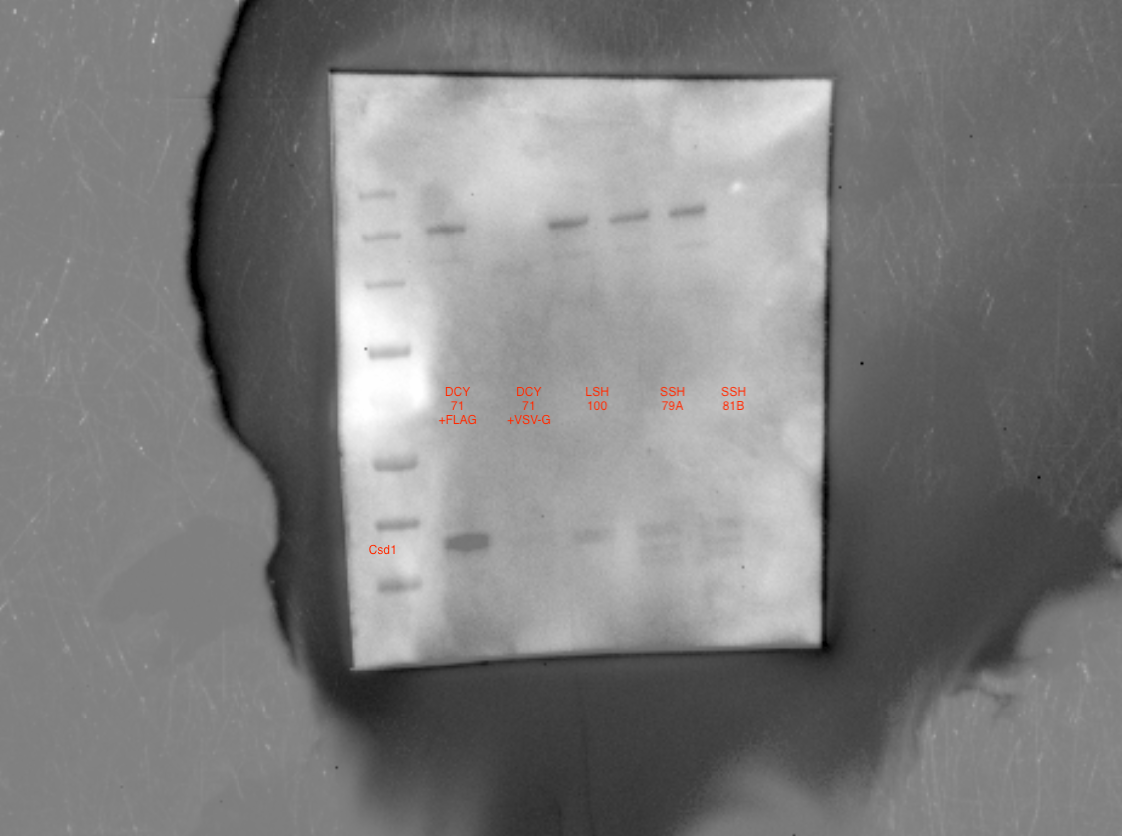

Supplement: Figure 6—source data 1. — In addition, raw, unedited Western blots probed with ɑ-CcmA and ɑ-FLAG, ɑ-Cag3 and ɑ-Csd1 antibodies to detect CcmA, Csd1, and Csd7-FLAG in co-immunoprecipitation experiments. [file elife-80111-fig6-data1.zip › Figure_6_source_data/Panel_C/IP_Csd1.tif]

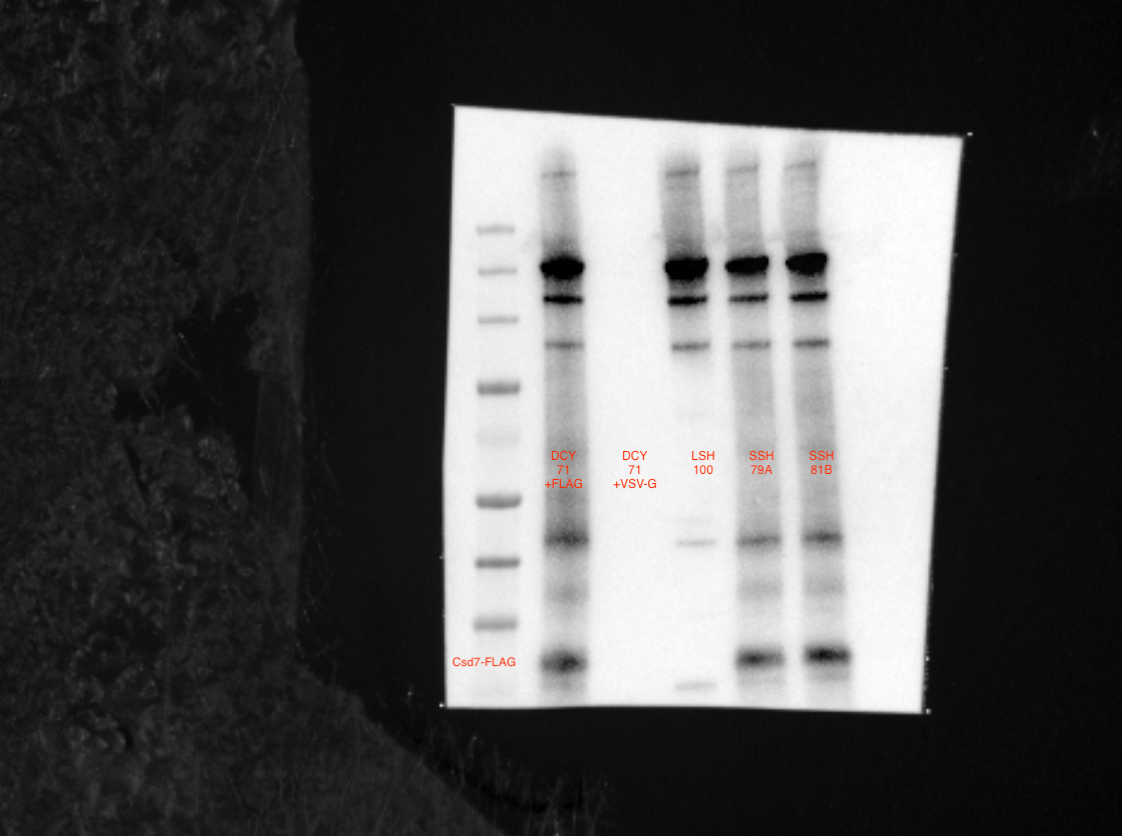

Supplement: Figure 6—source data 1. — In addition, raw, unedited Western blots probed with ɑ-CcmA and ɑ-FLAG, ɑ-Cag3 and ɑ-Csd1 antibodies to detect CcmA, Csd1, and Csd7-FLAG in co-immunoprecipitation experiments. [file elife-80111-fig6-data1.zip › Figure_6_source_data/Panel_C/IP_FLAG.tif]

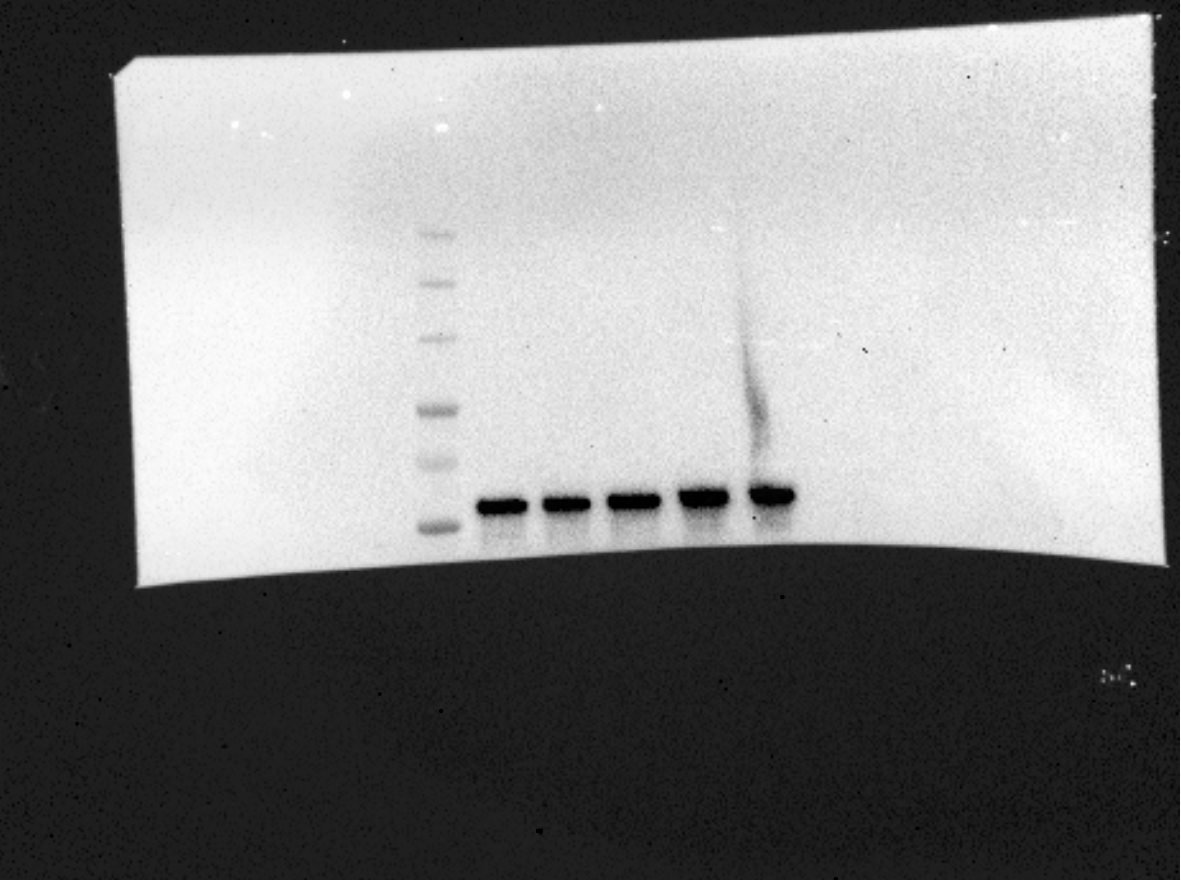

Supplement: Figure 7—figure supplement 2—source data 1. [file elife-80111-fig7-figsupp2-data1.zip › Figure_7_supp_2C_source_data/Cag3.tif]

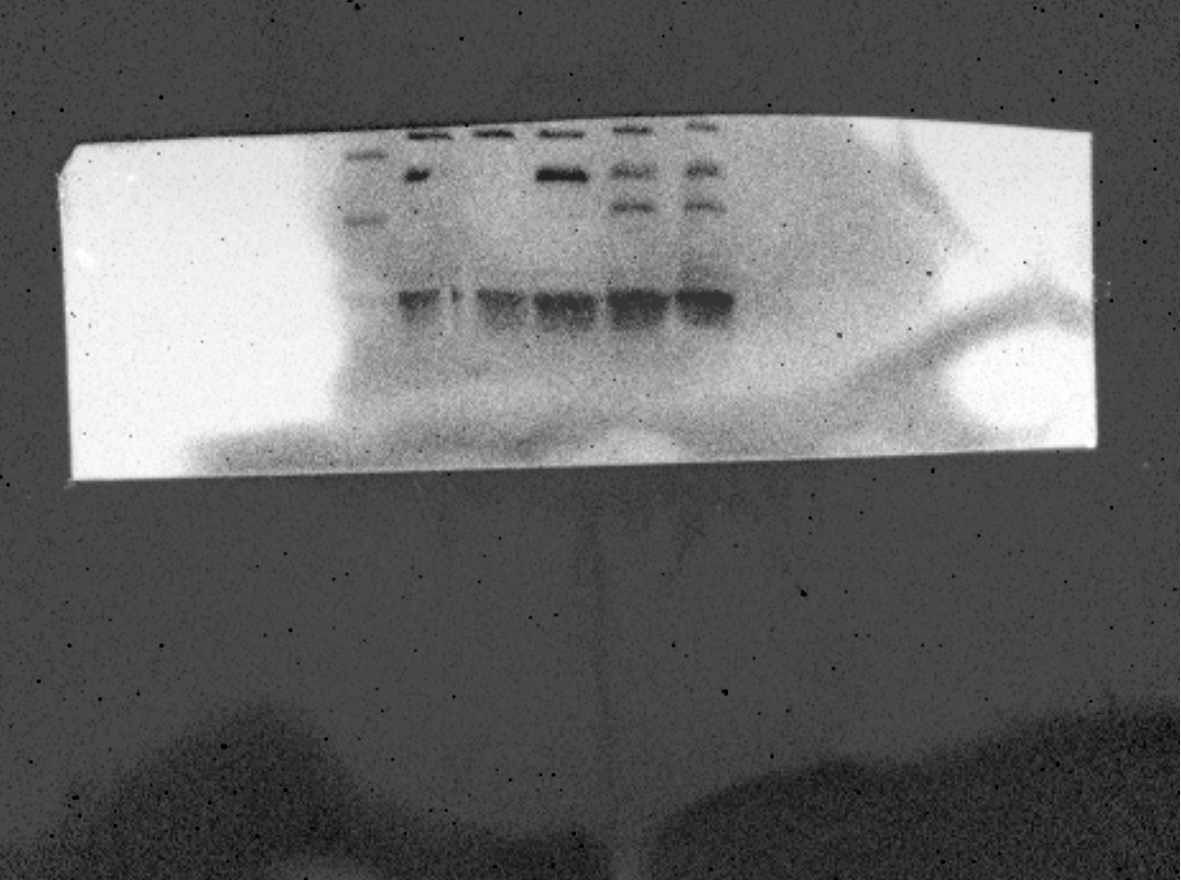

Supplement: Figure 7—figure supplement 2—source data 1. [file elife-80111-fig7-figsupp2-data1.zip › Figure_7_supp_2C_source_data/Csd1.tif]
